# RRCRank: a fusion method using rank strategy for residue-residue contact prediction

**DOI:** 10.1186/s12859-017-1811-9

**Published:** 2017-09-02

**Authors:** Xiaoyang Jing, Qiwen Dong, Ruqian Lu

**Affiliations:** 1 School of Computer Science, Fudan University, Shanghai, 200433 People’s Republic of China; 2 School of Data Science and Engineering, East China Normal University, Shanghai, 200062 People’s Republic of China

**Keywords:** Protein residue-residue contact prediction, Learning-to-rank, Fusion method

## Abstract

**Background:**

In structural biology area, protein residue-residue contacts play a crucial role in protein structure prediction. Some researchers have found that the predicted residue-residue contacts could effectively constrain the conformational search space, which is significant for de novo protein structure prediction. In the last few decades, related researchers have developed various methods to predict residue-residue contacts, especially, significant performance has been achieved by using fusion methods in recent years. In this work, a novel fusion method based on rank strategy has been proposed to predict contacts. Unlike the traditional regression or classification strategies, the contact prediction task is regarded as a ranking task. First, two kinds of features are extracted from correlated mutations methods and ensemble machine-learning classifiers, and then the proposed method uses the learning-to-rank algorithm to predict contact probability of each residue pair.

**Results:**

First, we perform two benchmark tests for the proposed fusion method (RRCRank) on CASP11 dataset and CASP12 dataset respectively. The test results show that the RRCRank method outperforms other well-developed methods, especially for medium and short range contacts. Second, in order to verify the superiority of ranking strategy, we predict contacts by using the traditional regression and classification strategies based on the same features as ranking strategy. Compared with these two traditional strategies, the proposed ranking strategy shows better performance for three contact types, in particular for long range contacts. Third, the proposed RRCRank has been compared with several state-of-the-art methods in CASP11 and CASP12. The results show that the RRCRank could achieve comparable prediction precisions and is better than three methods in most assessment metrics.

**Conclusions:**

The learning-to-rank algorithm is introduced to develop a novel rank-based method for the residue-residue contact prediction of proteins, which achieves state-of-the-art performance based on the extensive assessment.

**Electronic supplementary material:**

The online version of this article (10.1186/s12859-017-1811-9) contains supplementary material, which is available to authorized users.

## Background

In the research area of structural biology, de novo protein structure prediction is a long-standing challenge. The main aim of de novo protein structure prediction is to predict protein 3-dimensional structures by using their sequences. In the past, researchers have developed various methods (such as fragment-based assembly methods and molecular dynamics simulation methods) to model structures with lowest free energy for certain protein sequences. Based on this strategy, those methods have predicted some small protein structures accurately [1, 2]. However, due to the search spaces of large protein structures are very large, de novo protein structure prediction methods perform poorly on those large protein targets [3, 4]. These years, an alternative method is adopted to compress the scale of calculation by using protein contact constraints. This method first predicts protein residue-residue contacts from residue sequences, and then predicts protein tertiary structures by using those predicted contacts as constraints [5]. One study published in 2015 has shown the importance of contact that accurate topology-level modeling could be achieved by using long-range contacts [6]. By adding contact prediction module, some protein structure prediction methods have achieved improved performances [7]. In addition to the de novo protein structure prediction, the protein contacts are very useful in protein structure alignment [8, 9], protein model quality assessment [10–12] and drug design [13] etc.

In order to predict protein contacts accurately, related researchers have developed many methods since the 1990s. Generally, these methods could be classified into five kinds: correlated mutations methods, machine-learning methods, fusion methods, template-based methods and 3D model-based methods. Correlated mutations methods have been extensively studied for more than twenty years. The basic hypothesis of correlated mutations methods is, the substitutions of amino acid site should occur in pairs to keep the stability of protein structures. Researchers have use many mathematic methods to identify correlated substitution from multiple sequence alignments (MSAs) and they can be divided into two classes: the local statistical methods and the global statistical methods. The local statistical methods are based on statistical independence of residue pairs, such as mutual information [14, 15], correlation coefficient [16–20], observed minus expected square approaches [21–23], etc. In order to eliminate the indirect-coupling effects and phylogenetic bias from MSAs, some global statistical methods are employed later, such as maximum entropy model [24, 25], inverse covariance estimation [26], pseudo-likelihood maximization [27–30], etc. Machine-learning methods formulate the protein residue-residue contact prediction as a classification task (contact or non-contact) or a regression task (the contact probabilities of residue pairs). Many machine-learning algorithms are used to predict contact probabilities by learning from protein native structures, such as support vector machines [31–33], neural networks [34–38], random forest [39, 40] and hidden Markov models [41] etc. The input features of machine-learning methods usually include position-specific scoring matrix (PSSM), predicted solvent accessibility, predicted secondary structure, amino acid distributions, sequence length, residue position, etc. Fusion methods combine machine-learning methods and correlated mutations methods. Some fusion methods [42–44], which are also seen as machine-learning methods generally, take the outputs of correlated mutations methods as part of features and train machine-learning algorithms to predict contact probability. The other fusion methods [6, 45] score probabilities to be contacts by using correlated mutations and machine-learning methods separately, and then make a fusion of those scores by using preassigned weights. Template-based methods [46, 47] take homologous proteins those have known structures as templates to predict protein contacts, which is similar with the strategy used by template-based protein structure prediction. However, there are many proteins without homologous protein templates, so the template-based methods are not very useful to predict protein contacts. 3D model-based methods predict the protein structure and deduce contacts from the predicted structure. Considering that the protein contacts are mainly used for protein structure prediction, 3D model-based methods have limited use in most cases.

In general, protein residue-residue contact prediction is often seen as a classification task or a regression task. We present the RRCRank (Residue-Residue Contact prediction by learning-to-Rank) [48], which is a novel fusion method. In the RRCRank, the contact prediction is regarded as a ranking task and the contact probabilities of residue pairs are predicted by using learning-to-rank strategy. In information retrieval area, the learning-to-rank problems have been widely studied. In a typical information retrieval process, the input is a specific query and some relevant documents, and the output is the score of every document which represent the relevance of the document with the query. Among various machine learning methods, the learning-to-rank method is very powerful to solve information retrieval problem. It first learns ranking strategy by using machine learning algorithms from training data, and then ranks every document in the test set using the ranking strategy. Taking into account its good performance in information retrieval area, many bioinformatics tasks adopt learning-to-rank methods to deal with rank-related problems, such as biomedical document retrieval [49], protein model quality assessment [50],disease name normalization [51], etc. Here, we regard the contact prediction task as ranking task and use learning-to-rank method to solve it. The proposed fusion method, RRCRank [52], contains two phases. First, it uses correlated mutations methods and ensemble machine-learning classifiers to predict contact probabilities of residue pairs. Then, it makes a fusion of those predictions by using the learning-to-rank algorithm, which improves the contact prediction performance.

## Methods

### Protein contact definition and assessment metrics

The protein residue-residue contacts are specific substructures in protein tertiary structures. In general, residue-residue contact represents those residue pairs whose inter-residue distance is less-than a given threshold in tertiary structure. In this work, we adopt the same definition of contact in CASP (Critical Assessment of protein Structure Prediction): a residue pair will be regarded as a contact if the Euclidean distance of their Cβ atoms (Cα for GLY) is less than 8 Å [53].

Generally, according to the separation of residue pair along the protein sequence, there are three kinds of contacts: long-range contacts (the separation is greater or equal to 24), medium-range contacts (the separation is between 12 and 23) and short-range contacts (the separation is between 6 and 11). The contacts usually belong to the same secondary structure if their separation is less than 6 residues, so those contacts are usually not considered in contact prediction [53].

Under a certain contact definition, the goal of protein contact prediction is to classify residue pairs in the protein tertiary structure (contact or non-contact). In order to assessment the contact prediction methods, related works often use the precision (or accuracy) metric: precision = TP/(TP + FP), where TP represents the number of true positive samples and FP represents the number of false positive samples. In practice, contact prediction methods will score the probability to every residue pair or to a subset of possible residue pairs. In this study, we use the sets of Top 5, L/10 and L/5 scored predicted pairs to evaluate the proposed method, where L is the target protein sequence length.

### Datasets

In this work, The PDBSELECT dataset [54] is used as training dataset, which is also used by previous works: SVMSEQ [31] and R2C [45]. The pair-wise sequence identity of protein sequences from PDBSELECT dataset is lower than 25%, which means that protein sequences are non-homologous in the training dataset. We select 553 protein sequences and the residue numbers of those sequences range from 50 to 300. Previous study [55] has found the contacts are extremely sparse (~2–3%) in native tertiary structures, and it is the same in this training dataset. The sparse contacts distribution will lead to overtraining for non-contact samples, so when training the ensemble machine-learning classifiers, we sample training samples following the ratio of 4:1 between non-contact and contact samples.

To evaluate performances of the proposed method, the CASP11 (11th Community Wide Experiment on the Critical Assessment of Techniques for Protein Structure Prediction) [56] dataset and 40 CASP12 targets (55 domains) are used as test datasets. The sequence lengths of CASP11 dataset range from 44 to 669. The CASP11 dataset is divided into three categories (TBM, TBM-hard and FM) based on the official CASP definitions. In general, the protein targets of TBM-hard and FM categories are difficult to detect their homologous structure templates from known protein structures, so these protein targets are regarded as hard targets. The 40 CASP12 targets whose sequence lengths ranging from 75 to 670 were newly released in December 2016, and its targets and domains list are shown in Additional file 1: Table S1 and Additional file 2: Table S2. The release time of the training dataset is 2008, and the release time of CASP11 and CASP12 datasets are 2014 and 2016 separately, so the training dataset could not contain any targets in CASP11 and CASP12. For the hard targets in the test datasets, homologous structure template could not be find in the training dataset. More specifically, the average sequence similarity of all sequence pair is 12% and the proportion of sequence pairs those share more than 25% identity is 2.97% between PDBSELECT and CASP11 dataset. The highest sequence pair similarity is 44%, and there are just 16 sequence pairs’ (56,959 pairs in total) similarities in the range of 40%–45%. For PDBSELECT and CASP12 dataset, the average sequence similarity is 12% and the proportion of sequence pairs those share more than 25% identity is 2.97%. The corresponding highest sequence pair similarity is 44%, and there are 4 sequence pairs’ (22,120 pairs in total) similarities in the range of 40%–45%. These data demonstrate that the sequence similarity between train and test datasets is at a low level. In order to make a comprehensive assessment, we evaluate performances of the proposed method based CASP11 all targets and hard targets separately. It should be noted that we evaluate the performance of all methods based on protein domain (sequence length > 50), which is the common way adopted by most related studies. Additional file 3: Figure. S1 shows distributions of protein domains’ length on the CASP11 dataset and CASP12 dataset used in our work. Additional file 4: Figure. S2 shows the distributions of protein sequence similarity between train and test datasets.

### Contact prediction framework based on learning-to-rank

In machine learning research area, learning-to-rank is a kind algorithm that sorts objects based on their importance or relevance to the target by using a ranking strategy. In the past years, learning-to-rank algorithm has effectively solved many information retrieval problems, such as collaborative filtering [57], document retrieval [58], spam detection [59], etc. There are three categories of existing learning-to-rank algorithms: pointwise algorithm, pairwise algorithm, and list-wise algorithm. Different algorithms will handle the learning-to-rank process by using different strategies. Compared with the list-wise algorithm, the pointwise and pairwise algorithms have an obvious advantage that they could directly use the traditional classification or regression methodologies to the learning task. What’s more, pairwise algorithm usually outperforms pointwise algorithm and has been widely used in information retrieval applications.

For the pairwise algorithm, the learning-to-rank task is converted into a classification task. It represents each document as a feature vector and takes documents pairs as instances before learning period. The pairwise algorithm first collect document pairs from document lists, and then assign labels to represent relative importance or relevance of the two document for each document pair. Finally, it trains a machine learning model (regression or classification) with the labeled data. In test period, the trained rank model before is used to rank new data [58].

**Fig. 1:**
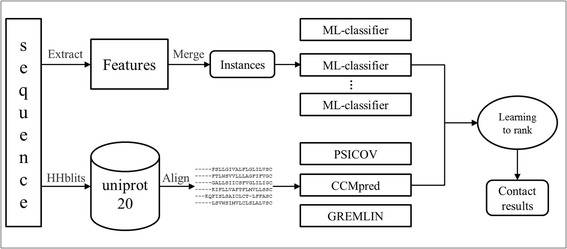
The overall flowchart of the proposed contact prediction framework

Referencing to the information retrieval strategy, the RRCRank also converts the contact prediction task to the ranking task. As shown in Fig. 1, the process can be divided into two phases: the RRCRank first uses correlated mutations methods and ensemble machine-learning classifiers to predict contact probability, and then it uses learning-to-rank method to re-rank each residue pair.

In first phase, the RRCRank uses three correlated mutations methods (CCMpred, PSICOV and GREMLIN) and ensemble machine-learning classifiers to predict the contact probability of residue pair. It is important to note that the ratio of contact samples in native protein structures is very low (~2–3%), which has also shown in previous study [55]. The imbalanced contact and non-contact distribution will lead to an extremely imbalanced learning problem. So we under-sample the non-contact residue pairs following the ratio of4:1 between non-contact and contact samples to deal with the imbalanced learning problem. Due to the under-sampling will cause information loss, we use ensemble machine-learning classifiers to counteract information loss. In this work, the classifier is random forest algorithm, so it is named as RF-classifier. For each contact type (long, medium and short), we under-sample three times and get three training subsets. The features are extracted from protein sequences, and a feature vector will represent a residue for the RF-classifier. In order to make use of local residue information, we adopt 9-residue window to represent the target residue, so an instance is an 18-residue window feature vector (a residue pair contains two residues). Then ensemble RF-classifiers take these instances as inputs to train the model or to predict unknown protein contacts. For correlated mutations methods (PSICOV, CCMpred and GREMLIN), the inputs of them is multiple sequence alignments (MSAs). Here we produce MSAs for each target protein sequence by using HHblits [60] against the uniprot20 database, then these MSAs are inputted into correlated mutations methods to make prediction. These correlated mutations methods will output decimals from 0 to 1 to represent contact probability, and a larger value means a greater contact probability in protein tertiary structure.

In the second phase, contact prediction task is converted to a ranking task by the RRCRank. In short, the RRCRank sorts residue pairs based on their contact probabilities in a certain protein structure. Based on the definition of contact, the Euclidean distance is used to measure the probabilities. The probability to be a contact of a residue pair would be large if their Euclidean distance is small. The learning-to-rank algorithm takes outputs of the first phase to train the ranking strategy, and then use the trained ranking strategy to score any target residue pairs for a protein to represent their relative ranking relation.

Here, the RRCRank adopts the pairwise ranking via-classification algorithm to predict contacts, and implement the task by using SVMRank [61]. It uses the linear kernel as kernel function and optimizes the parameters by using five-fold cross validation on the training dataset. Specifically, for a list of residue pairs of a target protein, we use the feature vector Φ(t, d) to represent the distance of t and d, where t is the target and d is the residue pair. We could get a list of ranking functions as:

$$ \left({d}_i,{d}_j\right)\in {f}_{\omega }(t)\iff \omega \cdot \varPhi \left(t,{d}_i\right)>\omega \cdot \varPhi \left(t,{d}_j\right) $$

where f is the ranking function, di and dj denote different residue pairs, and ω is the weight vector which is optimized in learning period.

Then, referring to SVM classification problem, slack variables are introduced and we could get the optimization problem as follow:

minimize:

$$ V\left(\omega, \xi \right)=\frac{1}{2}\omega \cdot \omega +C\sum {\xi}_{i,j,k} $$

subject to:

$$ {\displaystyle \begin{array}{c}\forall \left({d}_i,{d}_j\right)\in {r}_1:\omega \cdot \varPhi \left({t}_1,{d}_i\right)\ge \omega \cdot \varPhi \left({t}_1,{d}_j\right)+1-{\xi}_{i,j,1}\\ {}\cdots \\ {}\forall \left({d}_i,{d}_j\right)\in {r}_n:\omega \cdot \varPhi \left({t}_n,{d}_i\right)\ge \omega \cdot \varPhi \left({t}_n,{d}_j\right)+1-{\xi}_{i,j,n}\\ {}\forall i\forall j\forall k:{\xi}_{i,j,k}\ge 0\end{array}} $$

where V represents the objective function, C is a trading-off parameter of training error and margin size, ξ is the slack variable, k is the constraint subscript and r represents the residue pair set.

Next, a new optimization problem could be got by rearranging the constraints, which is an equivalent classification problem of SVM.

$$ \omega \cdot \left(\varPhi \left({t}_1,{d}_i\right)-\varPhi \left({t}_1,{d}_j\right)\right)\ge 1-{\xi}_{i,j,k} $$

According to the solution of SVM classification problem, we could also use decomposition algorithms to solve this problem.

### Feature extraction

There are two sets of features used in this study. The first set is various input features of RF-classifiers. We extract five types of sequence features for every residue: PSSM (Position Specific Scoring Matrix) and its relevant two outputs (relative weight of gapless real matches to pseudo-counts and information per position), predicted solvent accessibility, predicted secondary structure, Atchely factors and the residue relative position. The PSSM and its relevant outputs are obtained by running PSI-BLAST [62] on non-redundant sequence databases, here, we use the nr sequence database of NCBI which is filtered at 90 % sequence similarity as sequence database of PSI-BLAST, and run three iterations of PSI-BLAST for each target sequence. We use ACCpro and SSpro from SCRATCH [63] to predict solvent accessibility and secondary structure. The Atchely factors are five numerical values which represent volume, codon diversity, electrostatic charge, secondary structure and polarity [64], which characterize a residue by scaled representations. The relative position is calculated as: *rPosition = p/L*, where *L* is the protein sequence length and *p* is the target residue index.

The another feature set is the prediction values of three correlated mutations methods: PSICOV [26], CCMpred [65] and GREMLIN [29]. PSICOV is a representative correlated mutations method that uses the sparse inverse covariance estimation to predict inter-residue contacts [26]. The sparse inverse covariance estimation is a simple but powerful graphical inference technique to discriminate directly coupled mutation correlations from indirectly coupled correlations in the MSAs. CCMpred is a correlated mutations method by maximizing the pseudolikelihood of an L2-regularized Markov random field [65]. GREMLIN learns the direct couplings from a Markov random field by maximizing its pseudo-likelihood and incorporats prior information on pairs to be in contact to improve the robustness of predictions with fewer sequences [29].

## Results and discussion

### Performance improvements on CASP11 dataset

The contact prediction task is formulated as a ranking task by the proposed method, RRCRank. The RRCRank uses learning-to-rank method to sort each residue pair according to its contact probability. The inputs of the learning-to-rank algorithm are the predictions of three well-developed correlated mutations methods (CCMpred [65], PSICOV [26] and GREMLIN [29]) and machine-learning classifiers, and the outputs are floating values which represent relative ranking relations of residue pairs to be contacts. The method detail is shown in ‘Materials and Methods’ section. Each residue pair of a certain protein is ranked by the RRCRank based on its Euclidean distances from small to large, so the residue pair with higher ranking will have larger probability to be contact.

In order to evaluate the improvements made by the RRCRank, we performed a benchmark test on the CASP11 dataset. The assessment results based on CASP11 all targets are shown in Table 1. As shown in the table, the RRCRank clearly.

**Table 1:** The comparative results of the proposed method with other methods on CASP11 dataset

Methods^a^	Short-range			Medium-range			Long-range		
	Top 5	L/10	L/5	Top 5	L/10	L/5	Top 5	L/10	L/5
PSICOV	35.12%	24.59%	19.00%	34.47%	26.75%	21.82%	40.98%	33.12%	28.02%
CCMpred	40.00%	30.13%	22.60%	40.33%	31.66%	26.36%	43.90%	38.55%	33.51%
GREMLIN	40.33%	29.71%	22.80%	40.49%	32.19%	26.55%	43.25%	38.19%	33.64%
RF-classifiers^b^	62.76%	50.11%	42.18%	37.87%	31.69%	28.27%	25.41%	22.74%	19.85%
RRCRank	**67.48%**	**54.97%**	**46.02%**	**47.38%**	**37.87%**	**31.74%**	**48.69%**	**40.78%**	**34.77%**

^a^The best results are shown in bold font. ^b^The average of three independent RF-classifiers for each contact category

**Fig. 2:**
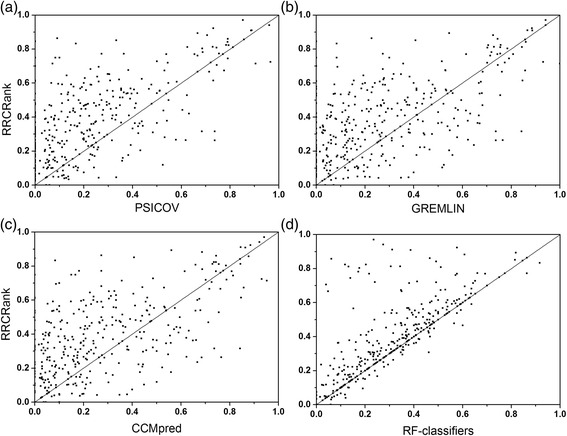
Comparison of the top L/5 prediction performance between the RRCRank and other methods. (**a**) PSICOV. (**b**) GREMLIN. (**c**) CCMpred. (**d**) RF-classifiers. (*Line* x = y is shown for reference)

shows a better performance for all three contact types, especially for short contacts and medium range contacts. In order to intuitively show the performance of RRCRank, the scatter plots of the prediction accuracy are shown on Fig. 2. As shown in the figure, most of targets are better predicted by the RRCRank, particularly compared with RF-classifiers.

Based on the design of RRCRank, two factors could contribute to its success. On the one hand, the learning-to- rank strategy could rank the residue pairs of a protein target structure reasonably. A protein structure can be seen as a micro-environment of its inner residue pairs, residue pairs from different protein structures could have different ranking relations even though they have similar elements. The learning-to-rank framework sorts residue pairs based on other residue pairs coming from the same structure, which is a more effective strategy than just giving global scores. One the other hand, the outputs of correlated mutations and ensemble machine-learning classifiers are complementary, the RRCRank achieves an improved performance by taking full advantage of these outputs as features. According to some previous researches, the performances of correlated mutations and machine-learning methods are different on different contact types, in Table 1, that phenomenon is also confirmed. For short-range contacts, the RF-classifiers achieves much better performance than correlated mutations methods (CCMpred, PSICOV and GREMLIN). However, for long and medium range contacts, correlated mutations methods achieves comparable even better performances, but the performance of RF-classifiers is not so well. Considering that the correlated mutations and machine-learning methods have complementary performances on different contact types, the integration of these two kinds of methods are valuable for predicting contact accurately. Due to learning-to-rank is a kind of machine-learning algorithm, the RRCRank achieves greater improvement for medium and short range contacts but less improvement for long range contacts.

Furthermore, we evaluate the improvements made by the RRCRank on CASP11 hard targets, and the results are shown in Additional file 5: Table S3. Because these hard targets are difficult to detect their homologous structure templates from known protein structures, it is a challenging task to predict their inter-residue contacts with high precision. As shown.

In Additional file 5: Table S3, the RRCRank shows better performances for medium and short range contacts when compared with other methods. And the proposed RRCRank achieves comparative performance for long-range contacts.

### Performance improvements on CASP12 dataset

In order to further evaluate the improvements made by the RRCRank on real data, we make a benchmark test on 40 CASP12 protein targets which were released in December 2016. The 40 CASP12 protein targets include 55 domains, in which 30 domains are hard targets, the detail is shown in Additional file 1: Table S1 and Additional file 2: Table S2. The evaluation results are shown in Table 2. As shown in the table, the overall performance of RRCRank is not better than CASP11 dataset. There could be two reasons. One is that most targets in CASP12 dataset are hard targets (~55%), while the ratio is approximate 41% on the CASP11 dataset. The other is that the CASP12 dataset contains more domains with long sequence length, which can be found from the distribution of protein domains’ length on these two datasets in Additional file 3: Figure. S1. However, similar to the results on CASP11 dataset, the RRCRank still has improved performances for three contact types in most metrics.

**Table 2:** The comparative results of the proposed method with other methods on CASP12 dataset

Methods^a^	Short-range			Medium-range			Long-range		
	Top 5	L/10	L/5	Top 5	L/10	L/5	Top 5	L/10	L/5
PSICOV	33.09%	25.99%	19.44%	38.55%	31.54%	23.86%	37.09%	33.65%	28.01%
CCMpred	40.00%	31.56%	24.10%	**46.91%**	36.52%	30.22%	41.82%	38.54%	**34.44%**
GREMLIN	40.00%	30.75%	24.08%	46.18%	35.84%	**30.25%**	44.00%	37.59%	34.31%
RF-classifiers	55.27%	45.78%	37.81%	31.64%	29.11%	23.67%	29.45%	23.04%	20.16%
RRCRank	**62.55%**	**51.59%**	**41.90%**	42.18%	**37.40%**	29.93%	**48.36%**	**39.34%**	34.37%

^a^The best results are shown in bold font

### Performance comparison with the regression-based and classification-based methods

Generally, most protein residue-residue contact prediction methods take classification or regression strategy to predict contacts, in this work, we propose a novel ranking strategy. In order to evaluate the superiority of ranking strategy, we implement a regression method (SVR) and a classification-based method (SVC) by using SVMlight [66]. The features fed into these two methods are same with those used in RRCRank. The regression-based method uses normalized Euclidean distance to score each residue pair, which is a similar way with that of RRCRank. The classification-based method takes the non-contacts as negative samples and contatcs as the positive samples.

Table 3 shows the assessment results. On the CASP11 all targets dataset, the proposed RRCRank outperforms the SVR and SVC methods except for the Top5 metric for short-range contacts. While on the CASP11 hard targets dataset, the RRCRank consistently outperforms the SVR and SVC methods on all assessment metrics.

**Table 3:** The comparative results of the proposed method with traditional strategies

Methods^a^		Short-range			Medium-range			Long-range		
		Top 5	L/10	L/5	Top 5	L/10	L/5	Top 5	L/10	L/5
All targets	SVR	**68.13%**	53.57%	44.98%	43.44%	36.20%	30.44%	38.37%	32.84%	27.96%
	SVC	62.60%	49.68%	42.26%	38.20%	32.05%	27.56%	36.89%	29.75%	26.51%
	RRCRank	67.48%	**54.97%**	**46.02%**	**47.38%**	**37.87%**	**31.74%**	**48.69%**	**40.78%**	**34.77%**
Hard targets	SVR	56.80%	45.83%	39.53%	35.20%	31.06%	26.07%	19.60%	16.40%	14.18%
	SVC	54.00%	44.77%	38.52%	35.60%	30.24%	26.09%	20.00%	15.62%	14.77%
	RRCRank	**57.20%**	**46.06%**	**39.72%**	**40.00%**	**31.39%**	**26.29%**	**30.40%**	**23.31%**	**18.57%**

^a^The best results are show with bold font for each category

For specific categories, there are significant improvements for long-range contacts made by the RRCRank, which indicates it has better prospects. Overall, the learning-to-rank method RRCRank is more competent for protein inter-residue contact prediction when compared with regression and classification methods.

### Performance comparison with four leading methods on CASP11 and CASP12 dataset

As an acknowledged assessment, the Critical Assessment of protein Structure Prediction (CASP) receives a great deal of attention by protein structure researchers. Groups with leading performance in CASP are recognized as the state-of-the-art methods in the corresponding period. To further assess the behavior of RRCRank, we select the best four methods (CONSIP2, Shen-Group, MULTICOM-CLUSTER and UCI-IGB-CMpro) in CASP11 [67] as references based on their L/5 precision (the definition is shown in ‘Materials and Methods’ section) for long-range contacts. In a sense, those four methods can be viewed as the best known methods these years. Among those four methods, the CONSIP2 is a fusion method which takes the outputs of correlated mutations methods as part of features and train a two-layer neural networks to predict contact probability, the Shen-Group is also a fusion method which makes a fusion of machine-learning and correlated mutations methods by using preassigned weights, the MULTICOM-CLUSTER is a machine-learning method based on deep networks and boosting techniques, and the UCI-IGB-Cmpro should be a machine-learning method based on deep neural networks (its category is inferred from the predictor’s article, which is not shown in CASP11 Abstracts).

**Fig. 3:**
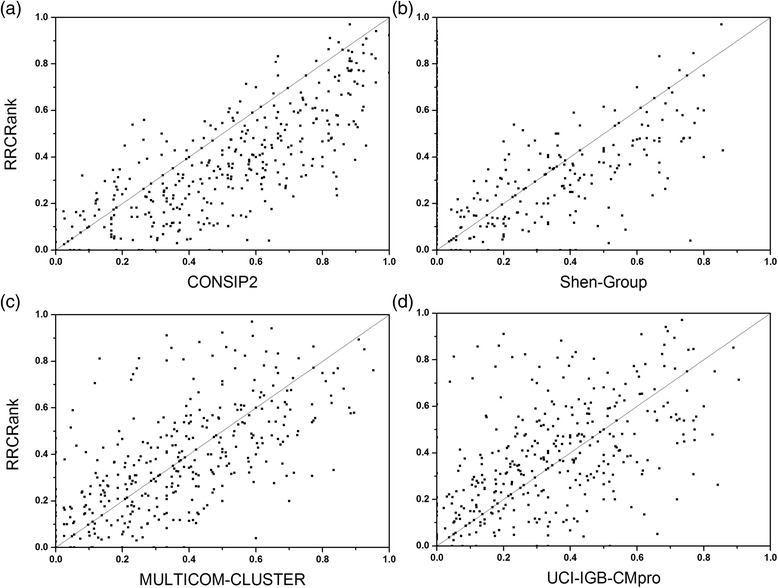
Comparison of the top L/5 prediction performance between the RRCRank and four leading methods in CASP11. (**a**) CONSIP2. (**b**) Shen-Group. (**c**) MULTICOM-CLUSTER. (**d**) UCI-IGB-CMpro. (Line x = y is shown for reference)

Table 4 shows the comparative results of RRCRank and the reference methods on the CASP11 dataset. Just as assessment results in CASP11 [67], the CONSIP2 is the top performing method which is superior to all other methods including the RRCRank. But when compared with other three leading methods, the RRCRank performs comparably, and on CASP11 all targets dataset, it outscores other three methods in most assessment metrics. In order to further demonstrate the value of RRCRank, we present the scatter plots of prediction accuracy comparison in Fig. 3. As shown in Fig. 3, the distribution of points is scattered, which means the RRCRank is not just a repeat of other methods but shows its superiority on some protein targets. What’s more, we select protein target T0817-D2 as an example to highlight the performance difference between the proposed RRCRank and four leading methods in Additional file 6: Figure. S3. In Fig. S3, real contacts are shown as grey dots, the contacts predicted by RRCRank are shown as black upper triangular in the upper left part of every subfigure, and the contacts predicted by four leading methods are shown as black down triangular in the lower right part of every subfigure. As shown in the figure, the contacts distribution predicted by RRCRank are different from those predicted by other leading methods. In general, those comparison results indicate that the RRCRank could achieve the state-of-the-art and unique performance for protein residue-residue contact prediction.

**Table 4:** The comparative results of the proposed method with the state-of-the-art methods on CASP11 dataset

Methods^a^		Short-range			Medium-range			Long-range		
		Top 5	L/10	L/5	Top 5	L/10	L/5	Top 5	L/10	L/5
All targets	CONSIP2	**75.77%**	**64.17%**	**55.18%**	**70.89%**	**59.91%**	**51.32%**	**58.37%**	**51.61%**	**46.65%**
	Shen-Group	58.31%	50.01%	43.11%	47.61%	41.10%	36.07%	34.37%	33.30%	28.94%
	MULTICOM-CLUSTER	68.13%	55.47%	46.17%	49.27%	41.12%	37.52%	35.12%	30.27%	26.32%
	UCI-IGB-CMpro	52.20%	42.79%	36.09%	48.94%	41.68%	36.09%	36.75%	30.38%	28.10%
	RRCRank	67.48%	54.97%	46.02%	47.38%	37.87%	31.74%	48.69%	40.78%	34.77%
Hard targets	CONSIP2	**68.40%**	**57.60%**	**50.48%**	**60.40%**	**50.22%**	**43.14%**	**41.60%**	**35.21%**	**30.36%**
	Shen-Group	60.89%	50.74%	43.72%	48.89%	41.62%	35.25%	29.33%	27.29%	22.94%
	MULTICOM-CLUSTER	62.40%	52.91%	43.81%	50.00%	40.31%	35.74%	24.40%	22.09%	17.89%
	UCI-IGB-CMpro	51.20%	42.58%	36.64%	49.20%	41.23%	34.94%	24.80%	19.93%	18.42%
	RRCRank	57.20%	46.06%	39.72%	40.00%	31.39%	26.29%	30.40%	23.31%	18.57%

^a^The best results are show with bold font for each category

Three of the four methods also participated in the CASP12, which are CONSIP2 (with the name MetaPSICOV in CASP12), Shen-Group and MULTICOM-CLUSTER. The comparison results of the RRCRank with these methods on 40 CASP12 targets are shown in Additional file 7: Table S4. As shown in table, the CONSIP2 (MetaPSICOV) performs best among these methods, but the RRCRank still has comparable performance compared with other methods.

### Statistical significance of difference between different methods

To estimate the degree of performance differences between RRCRank and other leading methods in CASP11, we use the *p*-values in Student’s t-test on prediction precision of different methods as the assessment metric. The results are presented in Table 5. We can find from the table, the *p*-values between RRCRank and other methods are very small, which means that differences are statistically significant. What’s more, we also calculate the *p*-values in Student’s t-test on prediction precision between the RRCRank and other methods used in the first phase on CASP11 and CASP12 datasets. The results are shown in Additional file 8: Table S5 and Additional file 9: Table S6. The differences are still statistically significant on CASP11 dataset. Though the CASP12 dataset is a small dataset, and the difference did not show in Additional file 9: Table S6 very significantly, it still reflects the approximate situation.

**Table 5:** The *p*-values in Student’s t-test for the difference on prediction precision between different methods on CASP11 dataset

Methods	CONSIP2	Shen-Group	MULTICOM-CLUSTER	UCI-IGB-CMpro	RRCRank
CONSIP2	1.00E + 00	1.28E-145	9.44E-36	1.15E-54	1.02E-27
Shen-Group	1.28E-145	1.00E + 00	1.53E-51	6.28E-34	1.37E-61
MULTICOM-CLUSTER	9.44E-36	1.53E-51	1.00E + 00	1.00E-03	1.00E-01
UCI-IGB-CMpro	1.15E-54	6.28E-34	1.00E-03	1.00E + 00	8.34E-07
RRCRank	1.02E-27	1.37E-61	1.00E-01	8.34E-07	1.00E + 00

Because correlated mutations methods are based on similar principle, *p*-values between PSICOV, CCMpred and GREMLIN are large. Empirically, in order to further improve the performance of the RRCRank, complementary correlated mutations methods could be more valuable. In summary, the head-to-head comparisons shows the proposed RRCRank has superiority compared with other methods.

## Conclusions

In structural biology area, protein residue-residue contacts are widely used. Especially in de novo protein structure prediction, conformational search space could be effectively constrained by residue-residue contacts. In this work, we present a contact prediction method RRCRank based on learning-to-rank, which solves the contact prediction task by using ranking strategy rather than traditional classification or regression strategy. First, the proposed method RRCRank uses correlated mutations methods and ensemble machine-learning classifiers to predict contact probabilities of residue pairs. Then, the RRCRank combines the complementary predictions of correlated mutations and machine-learning methods and uses the learning-to-rank method to make a fusion of those outputs, which improves the contact prediction performance. Benchmarked on CASP11 dataset and 40 CASP12 targets, improved performances on all three categories of contacts have been achieved by the proposed RRCRank, especially for medium and short range contacts. Compared with the classification and regression methods which use same features and processes, the proposed RRCRank shows a noteworthy superiority, especially for long-range contacts. Further, in order to make a more rigorous comparison, we select the best four methods in CASP11 as references and evaluation results indicate that the proposed RRCRank could achieve the state-of-the-art performance for protein residue-residue contact prediction.

The success of the RRCRank are contributed by two factors: the ranking strategy and the reasonable combination of complementary outputs of correlated mutations and machine-learning methods. A protein structure can be seen as a micro-environment of its inner residue pairs, therefore the ranking relations of residue pairs are affected by residue pairs from target protein. The proposed RRCRank scores a certain residue pairs by comparing it with other residue pairs from the target protein instead of just giving a global score, which is more appropriate for contact prediction task. Previous studies show that performances of correlated mutations and machine-learning methods are complementary for different contacts, which is also confirmed in this study. The proposed RRCRank could take advantage of those complementary predictions, which is another factor contributed to its success. To sum up, the RRCRank based on rank strategy could achieve the state-of-the-art performances. The RRCRank could be further improve its performance by introducing more complementary contact prediction methods.

## Additional files

Additional file 1: Table S1. Detailed list of the 40 protein targets of CASP12 dataset. (PDF 11 kb)

Additional file 2: Table S2. Detailed list of the 55 protein domains of CASP12 dataset. (PDF 30 kb)

Additional file 3: Figure. S1. The distributions of protein domains’ length on the CASP11 dataset and CASP12 dataset. (a) CASP11 dataset. (b) CASP12 dataset. (PDF 44 kb)

Additional file 4: Figure. S2. The distributions of protein sequence similarity between train and test datasets. (a) PDBSELECT and CASP11 dataset. (b) PDBSELECT and CASP12 dataset. (PDF 25 kb)

Additional file 5: Table S3. The comparative results of the proposed method with other methods on CASP11 hard targets. (PDF 15 kb)

Additional file 6: Figure. S3. Contact maps for proteins T0817-D2. (a) CONSIP2, (b) Shen-Group, (c) MULTICOM-CLUSTER and (d) UCI-IGB-CMpro. Real contacts are shown as grey dots, the contacts predicted by RRCRank are shown as black upper triangular in the upper left part of every subfigure, the contacts predicted by other methods are shown as black down triangular in the lower right part of every subfigure. (PDF 271 kb)

Additional file 7: Table S4. The comparative results of the proposed method with the state-of-the-art methods on 40 CASP12 targets. (PDF 13 kb)

Additional file 8: Table S5. The *p*-values in Student’s t-test for the difference on L/5 prediction precision between different methods on CASP11 dataset. (PDF 112 kb)

Additional file 9: Table S6. The *p*-values in Student’s t-test for the difference in L/5 prediction precision between different methods on CASP12 dataset. (PDF 258 kb)

## Abbreviations

CASP: Critical Assessment of protein Structure Prediction
CASP11: 11th Community Wide Experiment on the Critical Assessment of Techniques for Protein Structure Prediction
CASP12: 12th Community Wide Experiment on the Critical Assessment of Techniques for Protein Structure Prediction
RRCRank: Residue-Residue Contact prediction by learning-to-Rank

## References

[1] Lindorff-Larsen K, Piana S, Dror RO, Shaw DE (2011). How fast-folding proteins fold. Science.

[2] Bradley P, Misura KM, Baker D (2005). Toward high-resolution de novo structure prediction for small proteins. Science.

[3] Tai C-H, Bai H, Taylor TJ, Lee B: Assessment of template-free modeling in CASP10 and ROLL. Proteins-structure Function Bioinformatics 2014, 82 Suppl 2(Supplement S2):57–83. 10.1002/prot.24470 PMC7007176 24343678

[4] Piana S, Klepeis JL, Shaw DE (2014). Assessing the accuracy of physical models used in protein-folding simulations: quantitative evidence from long molecular dynamics simulations. Curr Opin Struct Biol.

[5] Marks DS, Hopf TA, Sander C (2012). Protein structure prediction from sequence variation. Nat Biotechnol.

[6] Ma J, Wang S, Wang Z, Xu J: Protein Contact Prediction by Integrating Joint Evolutionary Coupling Analysis and Supervised Learning. In: Research in Computational Molecular Biology: 2015. Springer: 218–221. 10.1093/bioinformatics/btv472 PMC4838177 26275894

[7] Zhang Y. I-TASSER: fully automated protein structure prediction in CASP8. Proteins Structure Function Bioinformatics. 2009;77(9):100–13. 10.1002/prot.22588 PMC2782770 19768687

[8] Wang S, Ma J, Peng J, Xu J: Protein structure alignment beyond spatial proximity. Sci Rep 2013, 3(3):1448–1448. 10.1038/srep01448 PMC3596798 23486213

[9] Xu J, Jiao F, Berger B (2007). A parameterized algorithm for protein structure alignment. J Comput Biol.

[10] Wang Z, Eickholt J, Cheng J (2011). APOLLO: a quality assessment service for single and multiple protein models. Bioinformatics.

[11] Miller CS, Eisenberg D (2008). Using inferred residue contacts to distinguish between correct and incorrect protein models. Bioinformatics.

[12] Tress ML, Valencia A. Predicted residue–residue contacts can help the scoring of 3D models. Proteins Structure Function Bioinformatics. 2010;78(8):1980–91. 10.1002/prot.22714 20408174

[13] Kliger Y, Levy O, Oren A, Ashkenazy H, Tiran Z, Novik A, Rosenberg A, Amir A, Wool A, Toporik A (2009). Peptides modulating conformational changes in secreted chaperones: from in silico design to preclinical proof of concept. Proc Natl Acad Sci.

[14] Korber BT, Farber RM, Wolpert DH, Lapedes AS (1993). Covariation of mutations in the V3 loop of human immunodeficiency virus type 1 envelope protein: an information theoretic analysis. Proc Natl Acad Sci U S A.

[15] Clarke ND (1995). Covariation of residues in the homeodomain sequence family. Protein Sci.

[16] Gobel U, Sander C, Schneider R, Valencia A (1994). Correlated mutations and residue contacts in proteins. Proteins-Structure Function and Genetics.

[17] Neher E (1994). How frequent are correlated changes in families of protein sequences?. Proc Natl Acad Sci.

[18] Taylor WR, Hatrick K (1994). Compensating changes in protein multiple sequence alignments. Protein Eng.

[19] Olmea O, Valencia A. Improving contact predictions by the combination of correlated mutations and other sources of sequence information. Folding design. 1997;2(3):25. 10.1016/s1359-0278(97)00060-6 9218963

[20] Pazos F, Helmer-Citterich M, Ausiello G, Valencia A (1997). Correlated mutations contain information about protein-protein interaction. J Mol Biol.

[21] Larson SM, Di Nardo AA, Davidson AR (2000). Analysis of covariation in an SH3 domain sequence alignment: applications in tertiary contact prediction and the design of compensating hydrophobic core substitutions. J Mol Biol.

[22] Kass I, Horovitz A (2002). Mapping pathways of allosteric communication in GroEL by analysis of correlated mutations. Proteins: Structure, Function, and Bioinformatics.

[23] Orly N, Miriam E, Amnon H. Detection and reduction of evolutionary noise in correlated mutation analysis. Protein Engineering Design Selection. 2005;18(5):247–53. 10.1093/protein/gzi029 15911538

[24] Lapedes AS, Giraud BG, Liu L, Stormo GD. Correlated mutations in models of protein sequences: phylogenetic and structural effects. Lecture Notes-Monograph Series. 1999:236–56.

[25] Weigt M, White RA, Szurmant H, Hoch JA, Hwa T (2009). Identification of direct residue contacts in protein–protein interaction by message passing. Proc Natl Acad Sci.

[26] Jones DT, Buchan DW, Cozzetto D, Pontil M (2012). PSICOV: precise structural contact prediction using sparse inverse covariance estimation on large multiple sequence alignments. Bioinformatics.

[27] Ekeberg M, Lövkvist C, Lan Y, Weigt M, Aurell E (2013). Improved contact prediction in proteins: using pseudolikelihoods to infer Potts models. Physical review E, Statistical, nonlinear, and soft matter physics.

[28] Feinauer C, Skwark MJ, Pagnani A, Aurell E (2014). Improving contact prediction along three dimensions. PLoS Comput Biol.

[29] Kamisetty H, Ovchinnikov S, Baker D (2013). Assessing the utility of coevolution-based residue–residue contact predictions in a sequence-and structure-rich era. Proc Natl Acad Sci.

[30] Balakrishnan S, Kamisetty H, Carbonell JG, Lee SI, Langmead CJ: Learning generative models for protein fold families. Proteins-structure Function Bioinformatics 2011, 79(4):1061&ndash;1078. 10.1002/prot.22934 21268112

[31] Wu S, Zhang Y (2008). A comprehensive assessment of sequence-based and template-based methods for protein contact prediction. Bioinformatics.

[32] Yuan Z (2005). Better prediction of protein contact number using a support vector regression analysis of amino acid sequence. BMC Bioinformatics.

[33] Cheng J, Baldi P (2007). Improved residue contact prediction using support vector machines and a large feature set. BMC bioinformatics.

[34] Shackelford G, Karplus K (2007). Contact prediction using mutual information and neural nets. Proteins.

[35] Punta M, Rost B (2005). PROFcon: novel prediction of long-range contacts. Bioinformatics.

[36] Xue B, Faraggi E, Zhou Y (2009). Predicting residue–residue contact maps by a two-layer, integrated neural-network method. Proteins: Structure, Function, and Bioinformatics.

[37] Fariselli P, Casadio R (1999). A neural network based predictor of residue contacts in proteins. Protein Eng.

[38] Tegge AN, Wang Z, Eickholt J, Cheng J (2009). NNcon: improved protein contact map prediction using 2D-recursive neural networks. Nucleic Acids Res.

[39] Li Y, Fang Y, Fang J (2011). Predicting residue-residue contacts using random forest models. Bioinformatics.

[40] Wang X, Chen Z, Wang C, Yan R, Zhang Z, Aguilar RC (2011). Predicting residue-residue contacts and helix-helix interactions in transmembrane proteins using an integrative feature-based random forest approach. PLoS One.

[41] Bjorkholm P, Daniluk P, Kryshtafovych A, Fidelis K, Andersson R, Hvidsten TR (2009). Using multi-data hidden Markov models trained on local neighborhoods of protein structure to predict residue-residue contacts. Bioinformatics.

[42] Wang Z, Xu J (2013). Predicting protein contact map using evolutionary and physical constraints by integer programming. Bioinformatics.

[43] Jones DT, Singh T, Kosciolek T, Tetchner S (2015). MetaPSICOV: combining coevolution methods for accurate prediction of contacts and long range hydrogen bonding in proteins. Bioinformatics.

[44] Kosciolek T, Jones DT. Accurate contact predictions using covariation techniques and machine learning. Proteins Structure Function Bioinformatics. 2015;84(S1):145–51. 10.1002/prot.24863 PMC5042084 26205532

[45] Yang J, Jin Q-Y, Zhang B, Shen H-B: R2C: Improving ab initio residue contact map prediction using dynamic fusion strategy and Gaussian noise filter. Bioinformatics 2016:btw181. 10.1093/bioinformatics/btw181 27153618

[46] Shao Y, Bystroff C (2003). Predicting interresidue contacts using templates and pathways. Proteins: Structure, Function, and Bioinformatics.

[47] Misura KM, Chivian D, Rohl CA, Kim DE, Baker D (2006). Physically realistic homology models built with ROSETTA can be more accurate than their templates. Proc Natl Acad Sci.

[48] Dong Q, Hu X (2017). RRCRank: a fusion method using rank strategy for residue-residue contacts prediction. Eur Biophys J.

[49] Wu J, Huang J, Ye Z. Learning to rank diversified results for biomedical information retrieval from multiple features. Biomed Eng Online 2014, 13 Suppl. 2:S3. 10.1186/1475-925X-13-S2-S3 PMC4304246 25560088

[50] Jing X, Wang K, Lu R, Dong Q (2016). Sorting protein decoys by machine-learning-to-rank. Sci Rep.

[51] Leaman R, Islamaj Dogan R, Lu Z (2013). DNorm: disease name normalization with pairwise learning to rank. Bioinformatics.

[52] Abstracts. Eur Biophys J. 2017;46(1):43–402.

[53] Monastyrskyy B, D'Andrea D, Fidelis K, Tramontano A, Kryshtafovych A (2014). Evaluation of residue-residue contact prediction in CASP10. Proteins.

[54] Hobohm U, Sander C (1994). Enlarged representative set of protein structures. Protein Sci.

[55] Bacardit J, Widera P, Márquez-Chamorro A, Divina F, Aguilar-Ruiz JS, Krasnogor N (2012). Contact map prediction using a large-scale ensemble of rule sets and the fusion of multiple predicted structural features. Bioinformatics.

[56] Kinch LN, Li W, Schaeffer RD, Dunbrack RL, Monastyrskyy B, Kryshtafovych A, Grishin NV (2016). CASP 11 target classification. Proteins Structure Function. Bioinformatics.

[57] Harrington EF. Online ranking / collaborative filtering using the perceptron algorithm. In: Proc of the Twentieth International Conference on. Mach Learn. 2003:250–7.

[58] Joachims T (2002). Optimizing search engines using clickthrough data. Proceedings of the eighth ACM SIGKDD international conference on knowledge discovery and data mining.

[59] Chirita P-A, Diederich J, Nejdl W: MailRank: using ranking for spam detection. In: Proceedings of the 14th ACM international conference on Information and knowledge management: 2005. ACM: 373–380.

[60] Remmert M, Biegert A, Hauser A, Söding J (2011). HHblits: lightning-fast iterative protein sequence searching by HMM-HMM alignment. Nat Methods.

[61] Joachims T (2006). Training linear SVMs in linear time. Proceedings of the 12th ACM SIGKDD international conference on knowledge discovery and data mining.

[62] Altschul SF, Madden TL, Schäffer AA, Zhang J, Zhang Z, Miller W, Lipman JD (1997). Gapped BLAST and PSI-BLAST: a new generation of protein database search programs. Nucleic Acids Res.

[63] Cheng J, Randall AZ, Sweredoski MJ, Baldi P (2005). SCRATCH: a protein structure and structural feature prediction server. Nucleic Acids Res.

[64] Atchley WR, Zhao J, Fernandes AD, Drüke T: Solving the protein sequence metric problem. Proceedings of the National Academy of Sciences of the United States of America 2005, 102(18):págs. 6395–6400. 10.1073/pnas.0408677102 PMC1088356 15851683

[65] Seemayer S, Gruber M, Soding J (2014). CCMpred--fast and precise prediction of protein residue-residue contacts from correlated mutations. Bioinformatics.

[66] Joachims T (1999). Making large scale SVM learning practical. Universität Dortmund.

[67] Monastyrskyy B, Dandrea D, Fidelis K, Tramontano A, Kryshtafovych A. New encouraging developments in contact prediction: assessment of the CASP11 results. Proteins-structure Function Bioinformatics. 2015;84(S1):131–44. 10.1002/prot.24943 PMC4834069 26474083

