# Supplementary material for: RRCRank: a fusion method using rank strategy for residue-residue contact prediction
Source: BMC Bioinformatics. 2017 Sep 2;18:390. doi: 10.1186/s12859-017-1811-9 (PMC5581475; doi:10.1186/s12859-017-1811-9)
Supplement: Supplementary file 1 — Detailed list of the 40 protein targets of CASP12 dataset. (PDF 11 kb) [file 12859_2017_1811_MOESM1_ESM.pdf]

Table S1. Detailed list of the 40 protein targets of CASP12 dataset

T0859	T0868	T0889	T0902	T0922
T0860	T0869	T0891	T0903	T0928
T0861	T0870	T0892	T0904	T0941
T0862	T0871	T0893	T0911	T0942
T0863	T0872	T0896	T0912	T0943
T0864	T0873	T0897	T0918	T0944
T0865	T0879	T0898	T0920	T0945
T0866	T0886	T0900	T0921	T0947
