# Supplementary material for: RRCRank: a fusion method using rank strategy for residue-residue contact prediction
Source: BMC Bioinformatics. 2017 Sep 2;18:390. doi: 10.1186/s12859-017-1811-9 (PMC5581475; doi:10.1186/s12859-017-1811-9)
Supplement: Supplementary file 2 — Detailed list of the 55 protein domains of CASP12 dataset. (PDF 30 kb) [file 12859_2017_1811_MOESM2_ESM.pdf]

Table S2. Detailed list of the 55 protein domains of CASP12 dataset

T0859-D1	FM	T0870-D1	FM	T0893-D1	TBM	T0903-D1	TBM	T0921-D1	TBM
T0860-D1	TBM	T0871-D1	TBM	T0893-D2	TBM	T0904-D1	FM	T0922-D1	TBM
T0861-D1	TBM	T0872-D1	TBM	T0896-D1	FM/TBM	T0911-D1	TBM	T0928-D1	TBM
T0862-D1	FM	T0873-D1	TBM	T0896-D2	FM/TBM	T0912-D1	TBM	T0941-D1	FM
T0863-D1	FM	T0879-D1	TBM	T0896-D3	FM	T0912-D2	FM/TBM	T0942-D1	TBM
T0863-D2	FM	T0886-D1	FM	T0897-D1	FM	T0912-D3	FM	T0942-D2	TBM
T0864-D1	FM	T0886-D2	FM	T0897-D2	FM	T0918-D1	FM	T0943-D1	FM/TBM
T0865-D1	TBM	T0889-D1	TBM	T0898-D1	FM	T0918-D2	FM	T0943-D2	TBM
T0866-D1	FM	T0891-D1	TBM	T0898-D2	FM/TBM	T0918-D3	FM	T0944-D1	TBM
T0868-D1	FM/TBM	T0892-D1	FM/TBM	T0900-D1	FM	T0920-D1	TBM	T0945-D1	FM/TBM
T0869-D1	FM	T0892-D2	FM	T0902-D1	TBM	T0920-D2	TBM	T0947-D1	TBM
