# Supplementary material for: RRCRank: a fusion method using rank strategy for residue-residue contact prediction
Source: BMC Bioinformatics. 2017 Sep 2;18:390. doi: 10.1186/s12859-017-1811-9 (PMC5581475; doi:10.1186/s12859-017-1811-9)
Supplement: Supplementary file 3 — The distributions of protein domains’ length on the CASP11 dataset and CASP12 dataset. (a) CASP11 dataset. (b) CASP12 dataset. (PDF 44 kb) [file 12859_2017_1811_MOESM3_ESM.pdf]

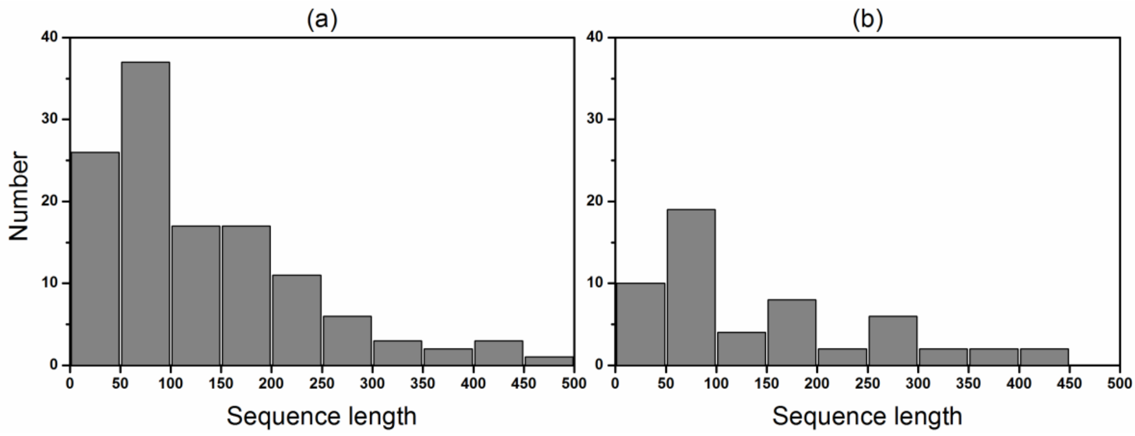

Fig. S1. The distributions of protein domains' length on the CASP11 dataset and CASP12 dataset. (a) CASP11 dataset. (b) CASP12 dataset.
