# Supplementary material for: RRCRank: a fusion method using rank strategy for residue-residue contact prediction
Source: BMC Bioinformatics. 2017 Sep 2;18:390. doi: 10.1186/s12859-017-1811-9 (PMC5581475; doi:10.1186/s12859-017-1811-9)
Supplement: Supplementary file 4 — The distributions of protein sequence similarity between train and test datasets. (a) PDBSELECT and CASP11 dataset. (b) PDBSELECT and CASP12 dataset. (PDF 25 kb) [file 12859_2017_1811_MOESM4_ESM.pdf]

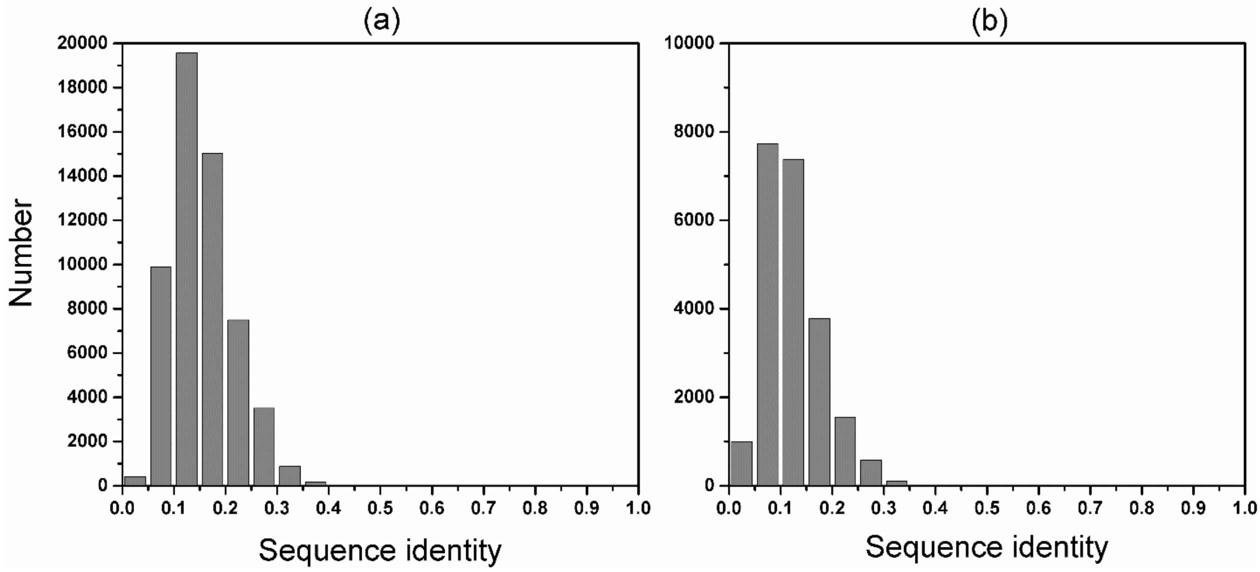

Fig. S2. The distributions of protein sequence similarity between train and test datasets. (a) PDBSELECT and CASP11 dataset. (b) PDBSELECT and CASP12 dataset.
