# Supplementary material for: RRCRank: a fusion method using rank strategy for residue-residue contact prediction
Source: BMC Bioinformatics. 2017 Sep 2;18:390. doi: 10.1186/s12859-017-1811-9 (PMC5581475; doi:10.1186/s12859-017-1811-9)
Supplement: Supplementary file 5 — The comparative results of the proposed method with other methods on CASP11 hard targets. (PDF 15 kb) [file 12859_2017_1811_MOESM5_ESM.pdf]

Table S3. The comparative results of the proposed method with other methods on CASP11 hard targets

Methods	Short-range			Medium-range			Long-range		
	**Top 5**	**L/10**	**L/5**	**Top 5**	**L/10**	**L/5**	**Top 5**	**L/10**	**L/5**
PSICOV	21.60%	14.86%	11.23%	18.80%	13.38%	10.76%	26.00%	19.05%	14.61%
CCMpred	26.80%	19.24%	14.98%	24.80%	16.86%	13.60%	**31.60%**	**26.95%**	21.29%
GREMLIN	28.00%	18.72%	15.30%	24.00%	17.80%	13.73%	30.80%	26.08%	**21.48%**
RF-classifiers[a]	54.40%	45.43%	38.40%	34.00%	29.45%	**26.90%**	13.60%	13.72%	12.35%
RRCRank	**57.20%**	**46.06%**	**39.72%**	**40.00%**	**31.39%**	26.29%	30.40%	23.31%	18.57%
