# Supplementary material for: RRCRank: a fusion method using rank strategy for residue-residue contact prediction
Source: BMC Bioinformatics. 2017 Sep 2;18:390. doi: 10.1186/s12859-017-1811-9 (PMC5581475; doi:10.1186/s12859-017-1811-9)
Supplement: Supplementary file 6 — Contact maps for proteins T0817-D2. (a) CONSIP2, (b) Shen-Group, (c) MULTICOM-CLUSTER and (d) UCI-IGB-CMpro. Real contacts are shown as grey dots, the contacts predicted by RRCRank are shown as black upper triangular in the upper left part of every subfigure, the contacts predicted by other methods are shown as black down triangular in the lower right part of every subfigure. (PDF 271 kb) [file 12859_2017_1811_MOESM6_ESM.pdf]

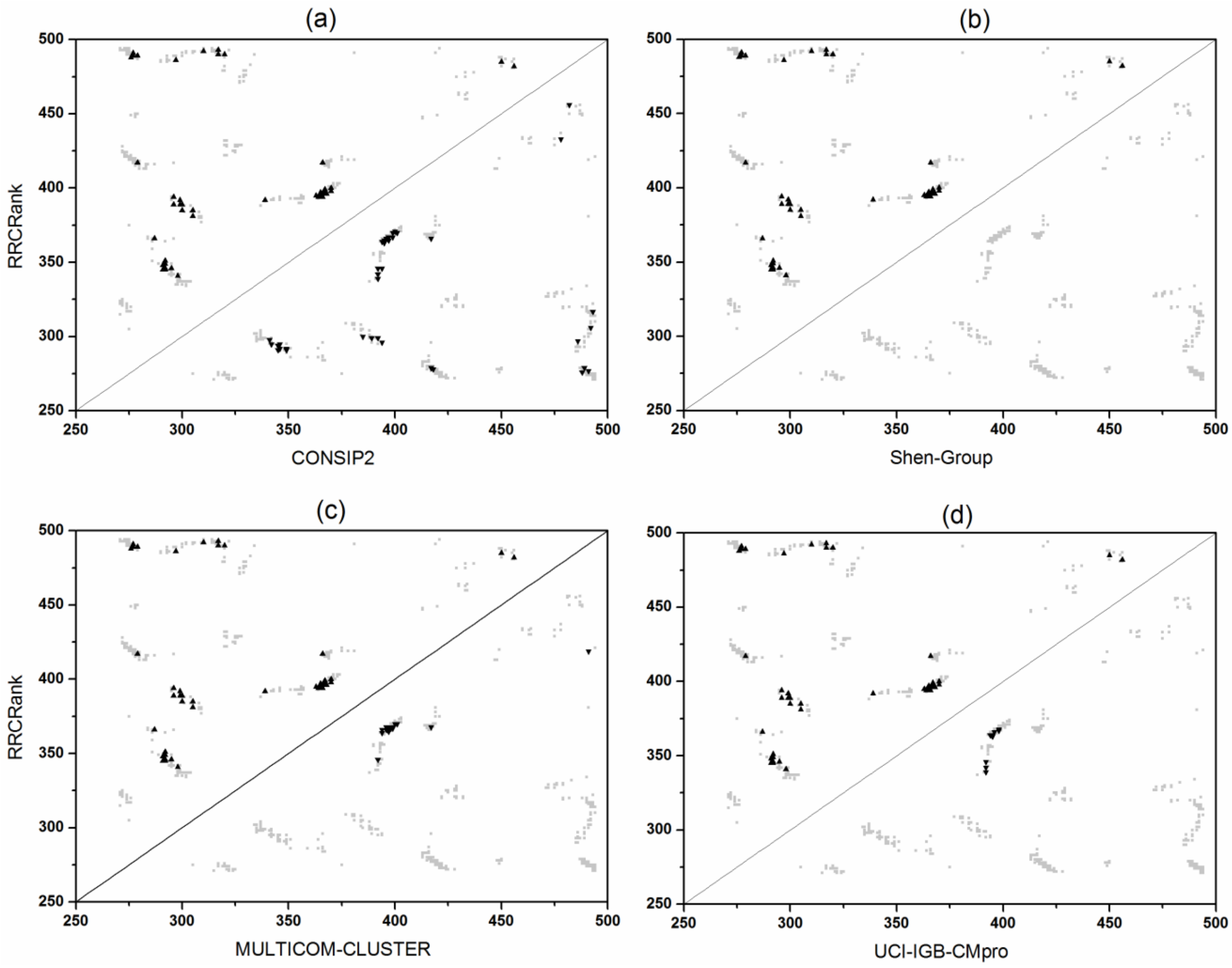

Fig. S3 Contact maps for proteins T0817-D2. (a) CONSIP2, (b) Shen-Group, (c) MULTICOM-CLUSTER and (d) UCI-IGB-CMpro. Real contacts are shown as grey dots, the contacts predicted by RRCRank are shown as black upper triangular in the upper left part of every subfigure, the contacts predicted by other methods are shown as black down triangular in the lower right part of every subfigure.
