# Supplementary material for: RRCRank: a fusion method using rank strategy for residue-residue contact prediction
Source: BMC Bioinformatics. 2017 Sep 2;18:390. doi: 10.1186/s12859-017-1811-9 (PMC5581475; doi:10.1186/s12859-017-1811-9)
Supplement: Supplementary file 7 — The comparative results of the proposed method with the state-of-the-art methods on 40 CASP12 targets. (PDF 13 kb) [file 12859_2017_1811_MOESM7_ESM.pdf]

Table S4. The comparative results of the proposed method with the state-of-the-art methods on 40 CASP12 targets

Methods	Short-range			Medium-range			Long-range		
	**Top 5**	**L/10**	**L/5**	**Top 5**	**L/10**	**L/5**	**Top 5**	**L/10**	**L/5**
MetaPSICOV	**70.18%**	**60.61%**	**52.61%**	**70.18%**	**63.00%**	**53.45%**	**65.45%**	**63.84%**	**60.44%**
Shen-Group	45.45%	39.26%	32.97%	40.73%	35.62%	29.71%	35.27%	30.09%	26.93%
MULTICOM-CLUSTER	18.55%	15.47%	13.28%	14.91%	14.40%	12.10%	58.91%	58.77%	54.50%
RRCRank	62.55%	51.59%	41.90%	42.18%	37.40%	29.93%	48.36%	39.34%	34.37%
