# Supplementary material for: RRCRank: a fusion method using rank strategy for residue-residue contact prediction
Source: BMC Bioinformatics. 2017 Sep 2;18:390. doi: 10.1186/s12859-017-1811-9 (PMC5581475; doi:10.1186/s12859-017-1811-9)
Supplement: Supplementary file 8 — The p-values in Student’s t-test for the difference on L/5 prediction precision between different methods on CASP11 dataset. (PDF 112 kb) [file 12859_2017_1811_MOESM8_ESM.pdf]

Table S5. The p-values in Student's t-test for the difference on L/5 prediction precision between different methods on CASP11 dataset

**Methods**	**PSICOV**	**CCMpred**	**GREMLIN**	**RF-classifiers**	**RRCRank**
PSICOV	1.00E+00	6.30E-02	5.57E-02	4.58E-04	1.69E-09
CCMpred	6.30E-02	1.00E+00	9.51E-01	1.96E-01	5.22E-05
GREMLIN	5.57E-02	9.51E-01	1.00E+00	2.25E-01	7.38E-05
RF-classifiers	4.58E-04	1.96E-01	2.25E-01	1.00E+00	5.91E-04
RRCRank	1.69E-09	5.22E-05	7.38E-05	5.91E-04	1.00E+00
