# Supplementary material for: RRCRank: a fusion method using rank strategy for residue-residue contact prediction
Source: BMC Bioinformatics. 2017 Sep 2;18:390. doi: 10.1186/s12859-017-1811-9 (PMC5581475; doi:10.1186/s12859-017-1811-9)
Supplement: Supplementary file 9 — The p-values in Student’s t-test for the difference in L/5 prediction precision between different methods on CASP12 dataset. (PDF 258 kb) [file 12859_2017_1811_MOESM9_ESM.pdf]

Table S6. The p-values in Student's t-test for the difference in L/5 prediction precision between different methods on CASP12 dataset

**Methods**	**PSICOV**	**CCMpred**	**GREMLIN**	**RF-classifiers**	**RRCRank**
PSICOV	1.00E+00	4.16E-02	4.63E-02	1.46E-01	1.34E-05
CCMpred	4.16E-02	1.00E+00	9.76E-01	3.43E-01	3.51E-02
GREMLIN	4.63E-02	9.76E-01	1.00E+00	3.65E-01	3.39E-02
RF-classifiers	1.46E-01	3.43E-01	3.65E-01	1.00E+00	3.24E-04
RRCRank	1.34E-05	3.51E-02	3.39E-02	3.24E-04	1.00E+00
